# Current controversies in infective endocarditis

**DOI:** 10.12688/f1000research.6949.1

**Published:** 2015-11-18

**Authors:** Thomas J. Cahill, Bernard D. Prendergast

**Affiliations:** 1 Department of Cardiology, Oxford University Hospitals, Oxford, UK; 2 Department of Cardiology, St Thomas's Hospital, London, UK

**Keywords:** Infective endocarditis, Bacteraemia, Antibiotic prophylaxis, Transcatheter valves, Multimodality imaging, Cardiac device infection

## Abstract

Infective endocarditis is a life-threatening disease caused by a focus of infection within the heart. For clinicians and scientists, it has been a moving target that has an evolving microbiology and a changing patient demographic. In the absence of an extensive evidence base to guide clinical practice, controversies abound. Here, we review three main areas of uncertainty: first, in prevention of infective endocarditis, including the role of antibiotic prophylaxis and strategies to reduce health care-associated bacteraemia; second, in diagnosis, specifically the use of multimodality imaging; third, we discuss the optimal timing of surgical intervention and the challenges posed by increasing rates of cardiac device infection.

## Introduction

In 1885, William Osler described ‘malignant’ endocarditis as an infection on the scarred heart valves of young adults with rheumatic heart disease ^1^. At the time, infective endocarditis (IE) was caused mainly by microorganisms originating in the oral cavity (oral streptococci). In 2015 in the developed world, IE looks dramatically different ^2^. Rheumatic fever is now rare, and in 25% of cases the causative infection is health care-acquired ^3, 4^. With the exception of intravenous drug users and those with congenital heart disease, patients typically are elderly. The focus of infection is frequently on prosthetic material within the heart: for example, cardiac devices (pacemakers, implantable cardioverter defibrillators and cardiac resynchronisation therapy) or prosthetic heart valves. In parallel, the bacteria underlying IE have changed: highly destructive staphylococci have now overtaken oral streptococci as the most common cause ^4, 5^. With an incidence of 3 to 10 per 100,000, IE is rare but carries an in-hospital mortality of 20% ^2^. Improving outcomes for patients with this complex and heterogeneous disease is challenging. In this review, we provide a focused update on current controversies in disease prevention, diagnosis and management.

## Prevention of infective endocarditis

Bacteria enter the bloodstream from the mouth in the course of daily life, and poor dental hygiene has long been recognised as a risk factor for IE ^6^. The significance of bacteraemia that occurs with dental extraction is still debated, as is the role of oral antibiotic prophylaxis to prevent it. In hospital, bacteraemia occurs as a complication of invasive procedures and indwelling venous catheters. Bacterial adherence, inflammation of the cardiac endothelium and thrombus deposition lead to formation of an infected ‘vegetation’ ^7^.

### Oral antibiotic prophylaxis

Poor oral hygiene is a risk factor for bacteraemia and subsequent IE ^8^. In addition to maintaining good hygiene, oral antibiotic prophylaxis has traditionally been prescribed for those at risk of IE prior to dental and surgical procedures. In 2008, the UK National Institute for Health and Care Excellence recommended against further use of prophylaxis, considering the potential hazards of widespread antibiotic use to outweigh the individual risks ^9^. The European Society of Cardiology (ESC) and American College of Cardiology/American Heart Association continued to recommend prophylaxis but only for patients at highest risk: those with previous IE, prosthetic valves and cyanotic congenital heart disease ^10^.

The effect of restricting antibiotic prophylaxis has now been examined in several studies ^11– 13^. Recently, a UK study found that over a 5-year period from 2008 there had been a small but significant increase in the (already-rising) number of IE cases alongside a fall in prescriptions for prophylaxis ^12^. These findings have been highly controversial. A causal link between IE cases and the withdrawal of systematic prophylaxis cannot be established by observational data, and the findings may be confounded by increased rates of bacteraemia or a growing at-risk population. Importantly, microbiological data were not available, so it is unclear whether the additional cases were caused by oral streptococci (the target of prophylaxis). In the absence of a randomised controlled trial (RCT)—which faces logistical, funding and ethical barriers—this study may tip the balance back toward prophylaxis for high-risk groups.

### Reducing health care-associated bacteraemia

At least a quarter of cases of IE are now acquired in the health-care setting ^14^. Mortality for health care-acquired IE exceeds 40% and this is due to the susceptible population, often with multiple comorbidities, and higher rates of *Staphylococcus aureus* infection ^15^. Health care-associated bacteraemia, the upstream cause of IE, is the target of several preventative strategies. Basic hand hygiene, aseptic technique, sterile barrier clothing and avoidance of the femoral route for venous catheters are all effective at reducing bacteraemia, but getting staff to adhere to best practice can be challenging ^16, 17^. ‘Practice-changing’ approaches, such as the use of a checklist to improve adherence to sterile technique, are powerful interventions which have been shown to reduce catheter-related infections ^18, 19^.

Infection during the implantation of cardiac devices is an important and preventable cause of IE and is reduced by use of perioperative antibiotics ^20^. Vaccination against *S. aureus*, which directly damages the cardiac endothelium to cause IE, is an attractive theoretical strategy ^21^. Unfortunately, a number of phase II and III trials have shown negative results, and the vaccine was associated with increased mortality in a 2012 study in patients undergoing cardiothoracic surgery ^22^. Other approaches in preclinical studies include novel materials designed to prevent bacterial adherence and agents that target bacterial biofilm production ^23, 24^.

## Diagnosis of infective endocarditis

Diagnosis of IE requires evaluation of clinical presentation, microbiology results and cardiac imaging ^2^. Reaching a definitive diagnosis can be challenging, particularly in suspected prosthetic valve IE and cardiac device infection (CDI), in which findings can be non-specific. The modified Duke criteria are useful for defining a positive diagnosis but were not intended as a clinical tool and cannot be rigidly applied to the individual patient ^25^. Strategies that facilitate earlier diagnosis, risk stratification and therapy are key to improving survival.

### Multimodality imaging for diagnosis of infective endocarditis

**Figure 1.**
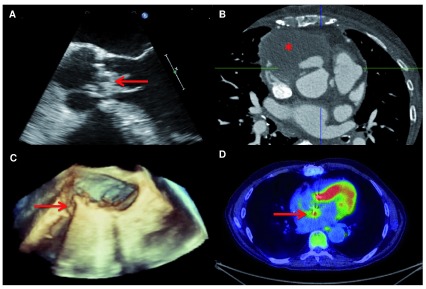
Multimodality imaging in diagnosis and detection of complications in infective endocarditis (IE). ( **A**) Echocardiography remains the core imaging modality in IE. Here, a vegetation (arrow) is visualised on the aortic valve by transoesophageal echocardiography. ( **B**) Computed tomography (CT) is excellent at defining the anatomical extent of complex endocarditis. A large paravalvular abscess (asterisk) can be seen complicating a case of prosthetic valve IE. ( **C**) Three-dimensional transoesophageal echocardiography provides a reconstructed view of the valve and here demonstrates dehiscence of a prosthetic mitral valve (arrow), an indication for surgical intervention. ( **D**) Positron emission tomography/CT has shown value for diagnosis of prosthetic valve IE and cardiac device infection. A focus of fludeoxyglucose- ^18^F ( ^18^F-FDG) uptake (arrow) can be seen at the site of a prosthetic aortic valve, separate from the myocardium, consistent with prosthetic valve IE. Adapted from Teoh *et al.* ^34^.

Transthoracic and transoesophageal echocardiography (TOE) are the mainstay of cardiac imaging for IE—for diagnosis, detection of complications and follow-up ( Figure 1A). Even after TOE, however, diagnosis is inconclusive in 10% to 20% of patients because of limited resolution or image artefacts ^26^, and adjunctive imaging techniques are showing promise in this group.

Cardiac computed tomography (CT) ( Figure 1B) is emerging as a valuable modality for detecting and defining IE complications in the aortic root (for example, formation of abscess/pseudoaneurysm) ^27, 28^. Identification of a paravalvular lesion (abscess, pseudoaneurysm or fistula) on cardiac CT is now a major criterion for the diagnosis of IE in the newly updated 2015 ESC IE guidelines ^29^. CT is less sensitive than transoesophageal echocardiography at detecting valvular perforations and may miss small vegetations, and interpretation can be challenging in the early postoperative period. Three-dimensional TOE can assist with detection of leaflet perforation and define the anatomy of valvular dehiscence ( Figure 1C) ^30, 31^.

^18^F-FDG-PET/CT (fludeoxyglucose- ^18^F/positron emission tomography/CT)—which shows uptake of radiolabelled glucose by metabolically active tissues—can visualise the infected vegetations of IE and has been successfully used in patients with suspected prosthetic valve IE or CDI, in which interpretation of TOE can be challenging ( Figure 1D) ^32– 34^. In a recently published study of 92 patients with suspected prosthetic valve IE or CDI, hybrid PET-CT enabled reclassification of 90% of patients (45 out of 50) with *possible* IE by Duke criteria and provided a conclusive diagnosis (definite/rejected) in 95% of cases ^35^. Addition of PET-CT to the Duke criteria raised the diagnostic sensitivity from 52% to 91%. As such, positive PET-CT (or single-photon emission computed tomography-CT) signal at the site of a prosthetic valve (at least 3 months after implant) has been included as a new major criterion for diagnosis of prosthetic valve IE in the 2015 ESC guidelines ^29^.

Beyond the heart, CT, magnetic resonance imaging (MRI) and PET imaging are facilitating early detection of embolism ^36^. Systematic MRI of the brain detects abnormalities in up to 80%, and imaging evidence of embolism is now a minor diagnostic criterion in the 2015 ESC guidelines ^37^. PET-CT imaging is useful for detection of peripheral vascular complications such as abscess, mycotic aneurysm and emboli ^35^. Given the range of imaging options now available, the next challenge is to define the optimal imaging strategy for specific patient subsets.

## Management of infective endocarditis

### The infective endocarditis team

Clinical care of patients with IE is complex and is reviewed in detail elsewhere ^2^. Management revolves around prolonged parenteral antibiotic therapy (typically 4 to 6 weeks in duration), surgical intervention for those at high risk or not responding to antibiotics, and surveillance for complications. Input is required from a wide range of specialists, including cardiologists, cardiothoracic surgeons, microbiologists and specialists in cardiovascular imaging and infectious diseases. Coordinating care can be a logistical challenge leading to delayed definitive management. Formation of a dedicated IE multidisciplinary team (MDT) is a simple strategy to improve clinical care. In a 2013 study from Italy, introduction of an IE MDT reduced in-hospital mortality from 28% to 13% ^38^. Recent UK guidelines advocate the formation of an IE team in every major centre ^39^.

### Early surgery

Surgery is currently performed in 40% to 50% of patients with IE ^40^. There are three main indications: valve dysfunction causing heart failure, uncontrolled infection, and prevention of embolism. Although seemingly clear-cut, the appropriateness and timing of surgery for an individual patient sometimes carry considerable uncertainty. In 2012, a landmark RCT compared early surgery (within 48 hours) with conventional treatment in stable patients with native valve IE, echocardiographically severe valvular regurgitation and large vegetations ^41^. The cohort was young (mean age of 47 years), was infected mainly by oral streptococci and had little comorbidity. The ‘early surgery’ group had a significant reduction in a composite endpoint of in-hospital death or embolism, driven by a reduction in the rate of embolism. This trial has led to a trend toward earlier surgery, but enthusiasm has been dampened by concerns that early surgery does not have the same benefits in the older, frailer IE population in much of the industrialized world. In prosthetic valve IE, retrospective studies looking at early surgery in patients have failed to find a benefit ^42, 43^.

Surgery to prevent embolism remains the most controversial indication, as the embolic risk for a specific individual is often difficult to predict. In general, surgery should be considered for those in the highest-risk groups: those with very large vegetations (>30 mm) or persistent large vegetations (>10 mm) after an embolic event and despite antibiotics ^29^. Mobile vegetations, staphylococcal infection and mitral valve location are also associated with increased embolic risk ^44^. The risk is highest in the first 2 weeks of antibiotic therapy and so intervention for prevention of embolism must be performed early to optimise the risk-to-benefit ratio of major surgery.

### Infective endocarditis on cardiac devices and transcatheter valves

The indications and implantation rates for cardiac devices have expanded dramatically in the last decade. Cardiac resynchronisation therapy is used widely for patients with advanced heart failure, and implantable (intracardiac) cardioverter defibrillators are commonly inserted for prevention of ventricular arrhythmias ^45^. Similarly, in the last 15 years, transcatheter aortic valve implantation (TAVI) has gone from concept to clinical reality, and over 100,000 valves have now been implanted worldwide ^46^. Cardiac device infection (CDI) currently accounts for roughly 10% of IE, and as use of cardiac devices and valve prostheses increases further, cardiologists should expect increasing rates of IE ^3^.

CDI affects 1% to 2% of patients in the first 5 years following device implantation and may involve the pocket housing the generator box, the leads or the endocardial surface ^20^. There are limited studies to direct clinical management of CDI, but UK guidelines outlining current best practice and recommendations for research have recently been published ^20^. Unless infection is superficial and limited to the healing wound, device extraction is required, and this can usually be achieved percutaneously but carries a small risk of mortality. The optimal duration of antibiotic therapy and risks of further infection after device re-implantation are unknown.

Reports of small cohorts of patients with IE following TAVI are starting to emerge ^47– 49^. A recently published multicentre registry identified a cohort of 53 patients with IE post-TAVI, representing a frequency of 0.5% at 1 year ^48^. The mean time to IE from TAVI procedure was 6 months. Interestingly, use of the self-expanding CoreValve system (Medtronic, Dublin, Ireland) was independently associated with increased risk of IE (hazard ratio 3.12, 95% confidence interval 1.37 to 7.14, *P* = 0.007). In-hospital mortality for this group was 47%, reflecting the high rates of staphylococcal infection and frailty of the study population. Further studies are required to better define the incidence, risk factors and clinical outcomes of IE following TAVI.

## Conclusions

IE is a rare and multifaceted disease whose heterogeneity is a barrier to well-powered research trials. As a consequence, much of the evidence base is derived from observational studies. Despite ongoing uncertainty, some conclusions can be drawn. Given the increasing use of intracardiac prostheses and devices, an ongoing focus on IE prevention strategies is warranted. Reducing time to diagnosis and definitive management requires set-up of IE clinical teams and full use of multimodality imaging alongside echocardiography. The importance of abnormal CT and hybrid PET-CT imaging is now reflected in the 2015 ESC guidelines on IE, in which abnormal imaging findings are novel diagnostic criteria. Finally, research networks should move toward a focus on multicentre trials, for example to address uncertainties in the timing of surgery and management of CDI.

## Abbreviations

CDI, cardiac device infection; CT, computed tomography; ESC, European Society of Cardiology; IE, infective endocarditis; MDT, multidisciplinary team; MRI, magnetic resonance imaging; PET, positron emission tomography; RCT, randomised controlled trial; TAVI, transcatheter aortic valve implantation; TOE, transoesophageal echocardiography.
